# Image‐based real‐time feedback control of magnetic digital microfluidics by artificial intelligence‐empowered rapid object detector for automated in vitro diagnostics

**DOI:** 10.1002/btm2.10428

**Published:** 2022-10-18

**Authors:** Yuxuan Tang, Fei Duan, Aiwu Zhou, Pojchanun Kanitthamniyom, Shaobo Luo, Xuyang Hu, Xudong Jiang, Shawn Vasoo, Xiaosheng Zhang, Yi Zhang

**Affiliations:** ^1^ School of Mechanical and Aerospace Engineering Nanyang Technological University Singapore Singapore; ^2^ Singapore Center for 3D Printing, School of Mechanical and Aerospace Engineering Nanyang Technological University Singapore Singapore; ^3^ School of Microelectronics Southern University of Science and Technology Shenzhen China; ^4^ China‐Singapore International Joint Research Institute Guangzhou China; ^5^ School of Electronic and Electrical Engineering Nanyang Technological University Singapore Singapore; ^6^ National Center for Infectious Disease Tan Tock Seng Hospital Singapore Singapore; ^7^ School of Electronic Science and Engineering University of Electronic Science and Technology of China Chengdu China

**Keywords:** artificial intelligence, In vitro diagnostics, magnetic digital microfluidics

## Abstract

In vitro diagnostics (IVD) plays a critical role in healthcare and public health management. Magnetic digital microfluidics (MDM) perform IVD assays by manipulating droplets on an open substrate with magnetic particles. Automated IVD based on MDM could reduce the risk of accidental exposure to contagious pathogens among healthcare workers. However, it remains challenging to create a fully automated IVD platform based on the MDM technology because of a lack of effective feedback control system to ensure the successful execution of various droplet operations required for IVD. In this work, an artificial intelligence (AI)‐empowered MDM platform with image‐based real‐time feedback control is presented. The AI is trained to recognize droplets and magnetic particles, measure their size, and determine their location and relationship in real time; it shows the ability to rectify failed droplet operations based on the feedback information, a function that is unattainable by conventional MDM platforms, thereby ensuring that the entire IVD process is not interrupted due to the failure of liquid handling. We demonstrate fundamental droplet operations, which include droplet transport, particle extraction, droplet merging and droplet mixing, on the MDM platform and show how the AI rectify failed droplet operations by acting upon the feedback information. Protein quantification and antibiotic resistance detection are performed on this AI‐empowered MDM platform, and the results obtained agree well with the benchmarks. We envision that this AI‐based feedback approach will be widely adopted not only by MDM but also by other types of digital microfluidic platforms to offer precise and error‐free droplet operations for a wide range of automated IVD applications.

## 1 INTRODUCTION

In vitro diagnostics (IVD) refers to medical tests that are done on biological samples extracted from bodily fluids or solid tissues, in order to detect the presence of pathogens, identify medical disorders, and monitor a person's health status or response to treatment.^1^ IVD plays an essential role in patient care and public health; billions of molecular IVD tests (PCR tests) and immunodiagnostic IVD tests (antibody tests) have been performed to contain global COVID‐19 pandemic.^2^, ^3^, ^4^, ^5^, ^6^ One of the riskiest factors involving IVD is sample handling, which may cause accidental exposure to contagious pathogens among healthcare workers. Automated IVD can reduce such risks by minimizing manual interventions required to handle hazardous biological samples.

Digital microfluidics provides an effective way of IVD automation.^7^, ^8^, ^9^, ^10^ Digital microfluidics is a type of liquid handling platform that manipulates microliter‐sized droplets on an open substrate. Digital microfluidic platforms employ droplets as reaction chambers for IVD, and handle the liquids by actuating these droplets that contain biological samples and assay reagents.^9^, ^10^, ^11^, ^12^ The droplet actuation mechanisms include magnetic force, electrowetting, and surface acoustic wave.^9^, ^13^, ^14^ Magnetic droplet actuation, hence the name magnetic digital microfluidics (MDM), attracts particular attention because it is relatively cost‐effective, straightforward to implement, and well‐suited to perform biological assays due to the bio‐affinity of magnetic particles used also for actuation.^9^, ^10^, ^15^, ^16^, ^17^, ^18^, ^19^ The pros and cons of electrowetting and magnetic droplet actuation are discussed in detail in Reference 9. However, it remains challenging to create a fully automated IVD platform based on the MDM technology because of a lack of effective feedback control system to ensure the successful execution of various droplet operations required for IVD. On MDM platforms, droplets are transported, merged and extracted via the added magnetic particles, and these droplet operations are a result of highly complex interactions that are influenced by the volume of droplets, quantity of particles, strength of magnetic field, moving speed, and surface tension of the substrate and liquids.^15^, ^20^ Currently, automated droplet manipulation on MDM platforms mainly relies on open‐loop control algorithms, assuming that the desired droplet operations could be successfully accomplished with a given set of pre‐determined parameters.^21^ However, these droplet operations fail at times due to surface imperfection, operating under borderline conditions, or inconsistent performance of the control equipment, but no mechanism is in place to rectify the failed operations.

Therefore, to create a fully automated IVD platform based on MDM, a closed‐loop feedback system must be in place to monitor droplet operations in real time and guide the control system to rectify the problems before moving on to the next step. So far, only a limited number of works on electrowetting‐based digital microfluidic platforms demonstrated the capability of feedback control by sensing the change in capacitive or resistive electrical signals when the droplets moved onto an electrode.^22^, ^23^, ^24^, ^25^, ^26^, ^27^ However, these sensing mechanisms require additional circuits, which increases the complexity and cost of the already complicated electrowetting‐based control system. Further, these sensing methods are not applicable to MDM because the substrate of MDM is free of electrodes due to the different actuation mechanism. On the other hand, imaging offers a way of contactless signal acquisition without the need to modify existing MDM platforms. The key challenge to implementing image‐based feedback for MDM is how to rapidly identify droplets and magnetic particles and determine the operation status based on this information. Conventional droplet detectors in electrowetting‐based digital microfluidic platform usually rely on Hough transform and edge detection algorithms.^28^, ^29^, ^30^ However, these algorithms are time‐consuming and ineffective in recognizing droplets on MDM platforms because the transparent water droplets do not show a strong contrast against the background and also do not always appear in regular shapes during movement. In contrast, artificial neural network (ANN) does not solely rely on edge features for object detection and has shown excellent performance in recognizing transparent droplets.^28^, ^29^, ^31^ Several closed‐channel emulsion droplet platforms used image feedback to control droplet generation and sorting by employing an ANN‐based object detector to identify droplets.^32^, ^33^, ^34^ Indeed, the ANN model for MDM needs to perform more complex tasks beyond just identifying droplets.

In this work, an artificial intelligence (AI)‐empowered MDM platform with image‐based real‐time feedback control is presented. An ANN object detector based on NanoDet,^35^ an ultrafast and lightweight target detection model, is trained to recognize droplets and magnetic particles, measure their size, and determine their location and relationship.^36^, ^37^, ^38^ This automated MDM platform shows the ability to rectify failed droplet operations based on the feedback information, ensuring that the entire IVD process is not interrupted due to the failure of liquid handling. Two IVD assays for protein quantification and antibiotic resistance detection are demonstrated on this platform, and both assays are fully automated to accomplish desired droplet operations without the need for human intervention in case of failure. To our best knowledge, this is the first smart MDM platform with AI‐empowered real‐time feedback, and we believe that this technology can be readily adopted by other types of digital microfluidic platforms to greatly broaden their applicability for IVD.

## 2 RESULTS AND DISCUSSION

### 2.1 AI‐empowered image‐based feedback control system for MDM

**FIGURE 1:**
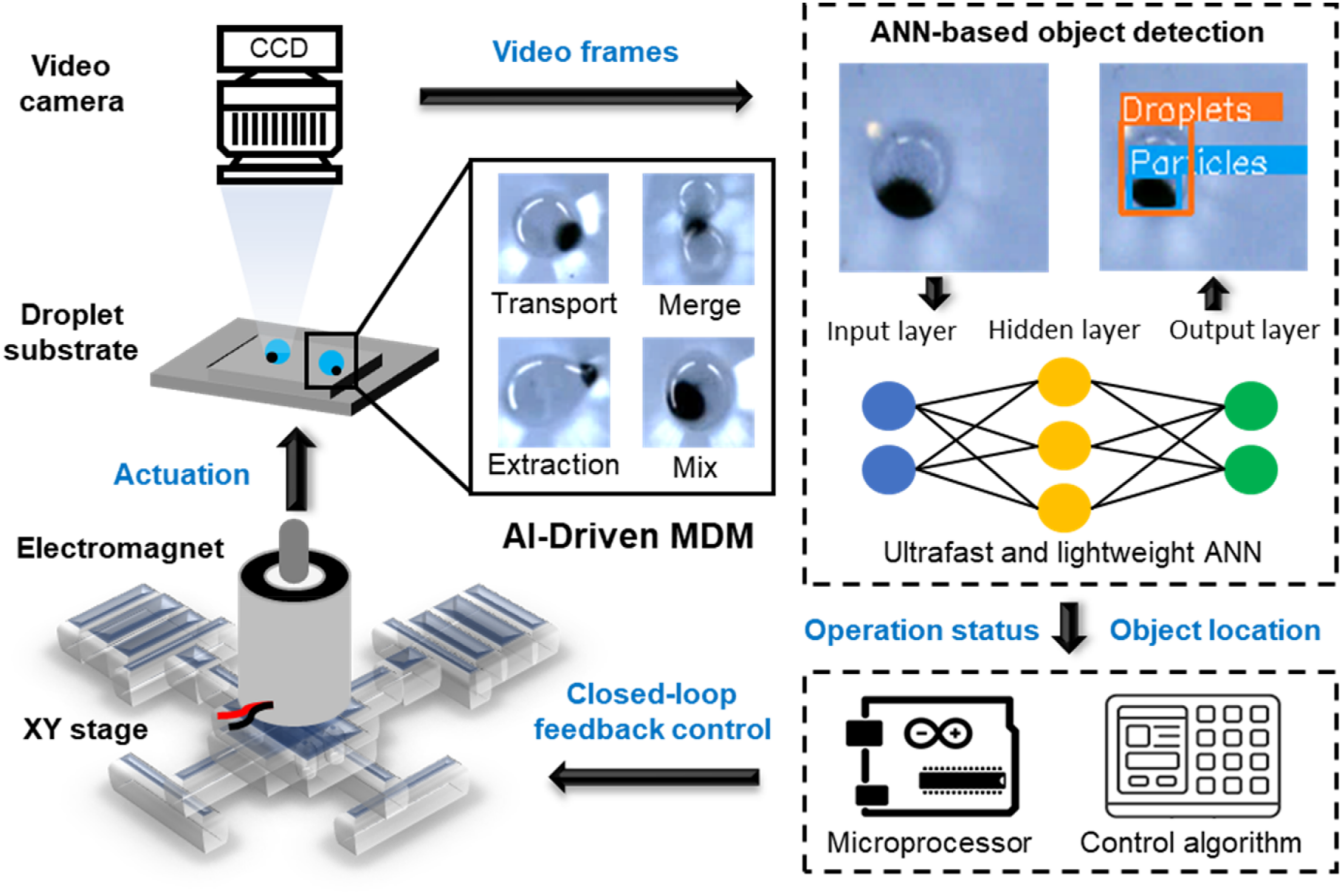
System overview of the AI‐empowered MDM platforms with image‐based real‐time closed‐loop feedback control.

The AI‐empowered MDM platform comprises an XY two‐axis translational stage with an electromagnet mounted on top, an MDM substrate for droplet manipulation, and a CCD camera that records the motion of the droplet and particles (Figure 1). The video frames are fed into an ultrafast and lightweight ANN model based on NanoDet that is trained to identify droplets and magnetic particles in the frames. Based on this information, a control algorithm tracks the location of the droplets and magnetic particles, judges the operation status, and checks if desired droplet operations are successfully accomplished. Commands are then issued by the algorithm to control the two‐axis translational stage and electromagnet via a microprocessor with closed‐loop feedback. One key feature of the feedback control is the ability to rectify failed droplet operations by moving back to the previous operation state and repeating the operation command.

**FIGURE 2:**
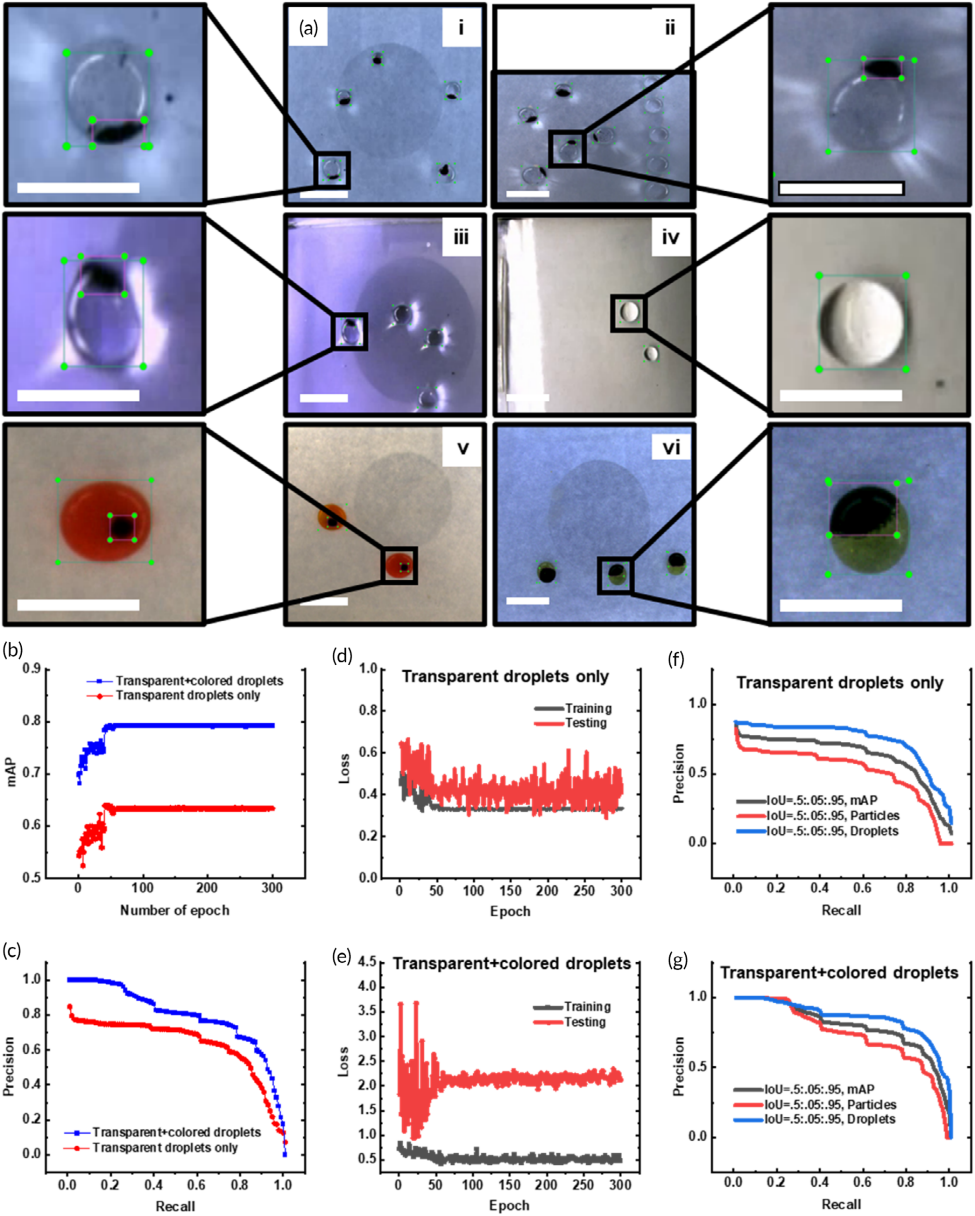
Training and evaluation of the ANN model for the feedback control of MDM. (a) Sample images with labeled objects used for training and testing; (b) mAP of the two trained models; (c) precision vs recall of the two trained models; (d) Loss versus epoch for the model trained with only transparent droplets; (e) Loss versus epoc for the model trained with both transparent and colored droplets; (f) precision versus recall curve of the model trained with only transparent droplets, and (g) precision versus recall curve of the model trained with both transparent and colored droplets. All scale bars represent 5 mm.

The ANN‐enabled object detector is trained based on NanoDet, a one‐stage anchor‐free object detection model with a model size of only 1.5 Mb and a latency of ~10 ms when implemented on Kirin 980 (4xA76 + 4xA55) ARM CPU based on NCNN.^35^ In our case, the latency time is 15.82 ms when implemented on Intel® Core™ i5‐8265U CPU @ 1.60 GHz. To train the ANN model, a total of 769 frames extracted from video recordings, which contained 3647 labels, were split 70%/30% into the training data set (538 frames with 2604 labels) and testing data set (231 frames with 1043 labels). These data sets contained six sets of labeled images (Figure 2a): (i) images with droplets of various particle/droplet (P/D) ratios; (ii) images of different aspect ratios containing droplets with or without particles; (iii) images extracted from videos wherein particles constantly move in and out of droplets; (iv) images of droplets under different illumination conditions; (v) images with colored droplets that contained reagents for the Carba NP assay for the detection of antibiotic resistance; and (vi) images with colored droplets that contained reagents for BCA assay for protein quantification. A total of two classes of objects, namely droplets and particles, were defined. Each object in the images, either a droplet or a magnetic particle cluster (appeared as a black dot), was labeled with an individual bounding box for supervised training (Figure 2a zoomed view).

Two versions of the ANN object detector were trained, and their performance was compared. The first version was trained with data from the first four sets of images which contained images of particles and only transparent droplets while the second version was trained with all six sets of images which included colored droplets. The performance of the two models was evaluated with the mean average precision (mAP) as per COCO definition (average precision over all classes at IoU = 0.50:0.05:0.95). The mAP of both models showed high variability before the 40th epochs; the variability drastically decreased after that, and the mAP remained almost a constant from the 60th to 300th epoch (Figure 2b). The first model trained with only transparent droplets showed a mAP value of ~0.63 while the second model showed a significantly higher mAP of ~0.8, possibly due to the ease of recognizing colored droplets. The precision versus recall curve showed that the second model trained with both transparent and colored droplets was better skilled at recognizing and discriminating target objects, i.e., droplets and particles in our case, in the images (Figure 2c). The loss of the first model had a large value and showed a large discrepancy between the testing and training data set (Figure 2d). In comparison, the loss of the second model had a small value and showed a small discrepancy (Figure 2e). This result again proved that the second model was better skilled at recognizing droplets and particles.

It was noticed that the particles had a lower mAP than the droplets in both models, evidenced by the smaller area under the curve of the precision versus recall curves for each class (Figure 2f,g) possibly due to the following two reasons: first, the particles appeared as a tiny dot in the images when they were present in a small quantity, which may be overshadowed by the reflection and refraction patterns of the droplets; second, the particles may spread and occupy the entire bottom surface of the droplet when they were present in a large quantity, in which case the droplets may also be identified as particles.

The second ANN model was hence selected for the feedback control of droplet operations on the MDM platform. The entire AI‐empowered MDM setup was situated in a soft‐tent light studio equipped with three strips of intensity‐adjustable LED illumination source (Figure S2a). The ANN‐based detector recognized droplets and particles under a dim illumination of 25 lux which was too dim for unaided eyes to see (Figure S2b). The setup was able to operate under a wide range of illumination conditions (25–1000 lux). This is a useful feature because different IVD assays require different lighting conditions, and many of them are preferably performed in an enclosure with dim lighting. Because of different lighting and imaging conditions, the background of the images may appear in different color and brightness. Regardless of the lighting and imaging conditions, our AI could identify the targets and manipulate them with high confidence. This reflects the strong generalizability of our platform.

### 2.2 Fundamental MDM operations with AI‐empowered feedback control

Four fundamental droplet operations on the MDM platform, which includes droplet transport, particle extraction, droplet merging, and mixing, are used to validate the AI‐empowered feedback control. A graphic user interface (GUI) is built to facilitate the user input and display droplet operations (Figure S2). The GUI displays two live streams of the entire field of view—one with the prediction labels and one without. The size and coordinates of the object, real‐time position of the control point (CP), motor speed and moving direction, and user‐specified locations are also displayed in the GUI. Users can click on the live stream to specify destinations for droplets, type in other operation parameters, such as direction and speed for movement, and reset the process. Please see supplementary information for the pseudocode of the functions described below.

#### 2.2.1 Droplet transport

**FIGURE 3:**
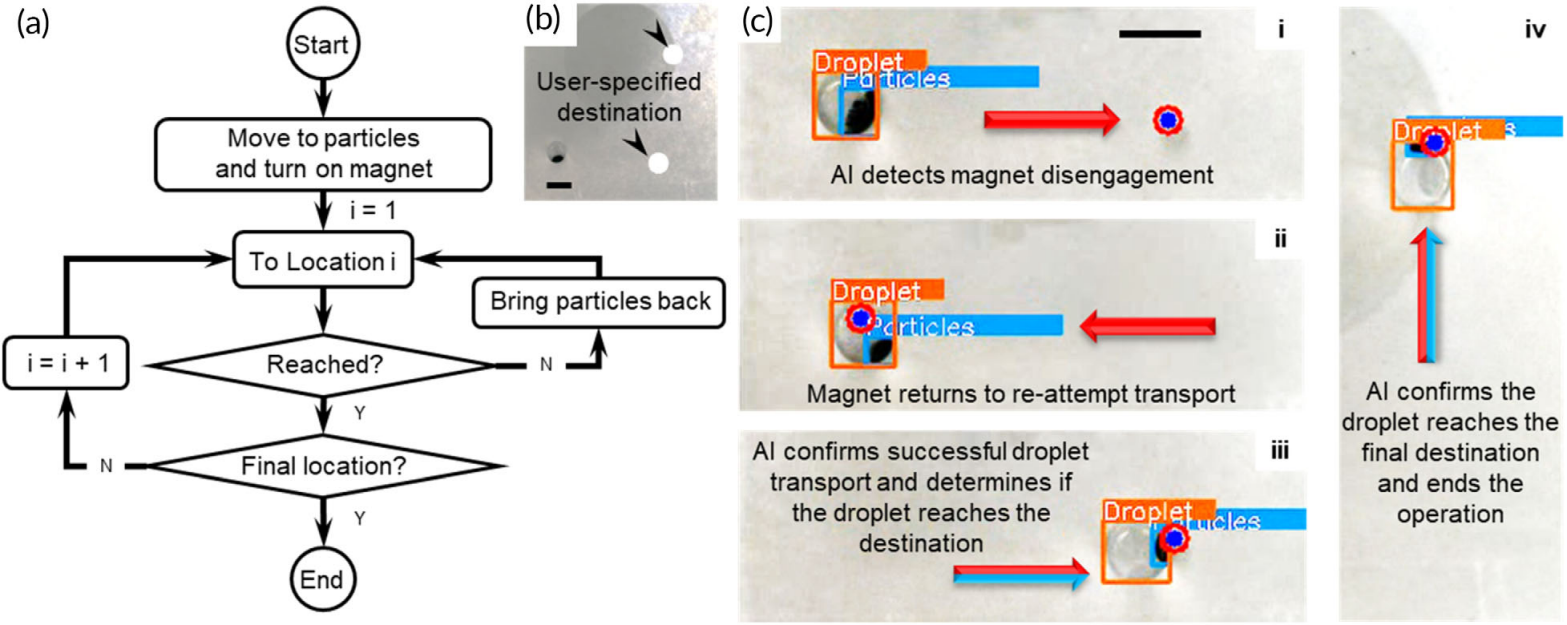
Droplet transport with AI‐empowered image‐based feedback for real‐time closed‐loop control. (a) The flowchart of the control algorithm for droplet transport. (b) A sample image of user‐specified destinations. (c) Droplet transport with AI‐enabled feedback control. All scale bars represent 5 mm.

The control algorithm for droplet transport is shown in the flowchart in Figure 3a. Before starting the operation, users specify the destinations by clicking in the top live stream area, and the destination would appear as a white circle in the bottom live stream (Figure 3b). Once started, the AI identifies the droplets and particles and determines their locations on the platform (Figure 3c and Video S1). The CP, which appears as a blue dot with a red outline in the images, moves to where the particles are located and switches on the electromagnet. There is about 1‐s delay between the CP and actual location of the electromagnet because of the response time of the hardware. Next, the electromagnet follows the CP and drags the particles to the first user‐specified destination at a default speed of 8.33 mm/s. In this process, the particles are trapped within the droplet due to the surface tension and bring the droplet to move along with them. However, if the quantity of the particles is too small and the moving speed is too high, the magnet may disengage from the particles, which means the particles and droplet cannot follow the movement of the magnet (Figure 3ci).^20^ The magnet disengagement leads to failed droplet transport, and hence human intervention is required to reset the operation in conventional MDM platforms. With AI‐empowered closed‐loop feedback, the platform detects the magnet disengagement by comparing the coordinate of the droplet with that of the user‐specified destination. If the droplet does not reach the destination, the CP guides the electromagnet back to where the particles are and restarts the droplet transport operation at a lower speed (Figure 3cii). Once the droplet reaches the first destination, the AI confirms the successful completion of the droplet transport operation before continuing with the next operation (Figure 3ciii). The entire operation ends when the AI confirms that the droplet has reached the final destination (Figure 3civ). During droplet transport, the magnet is at the leading edge of the droplet. An “overshoot” function is programmed to allow the particles to move beyond the destination along the y‐direction by 20 pixels and then move back by the same distance in order to position the center of the droplet and particles close to the coordinate of the destination, which could facilitate subsequent operations. This demonstration proves that the ANN object detector is able to identify droplets and particles and monitor their locations in real time for feedback control. Based on this feedback information, the AI judges whether the droplet transport operation is successful and commands the electromagnet to repeat the operation in the case of magnet disengagement to rectify the failed operation.

#### 2.2.2 Particle extraction

**FIGURE 4:**
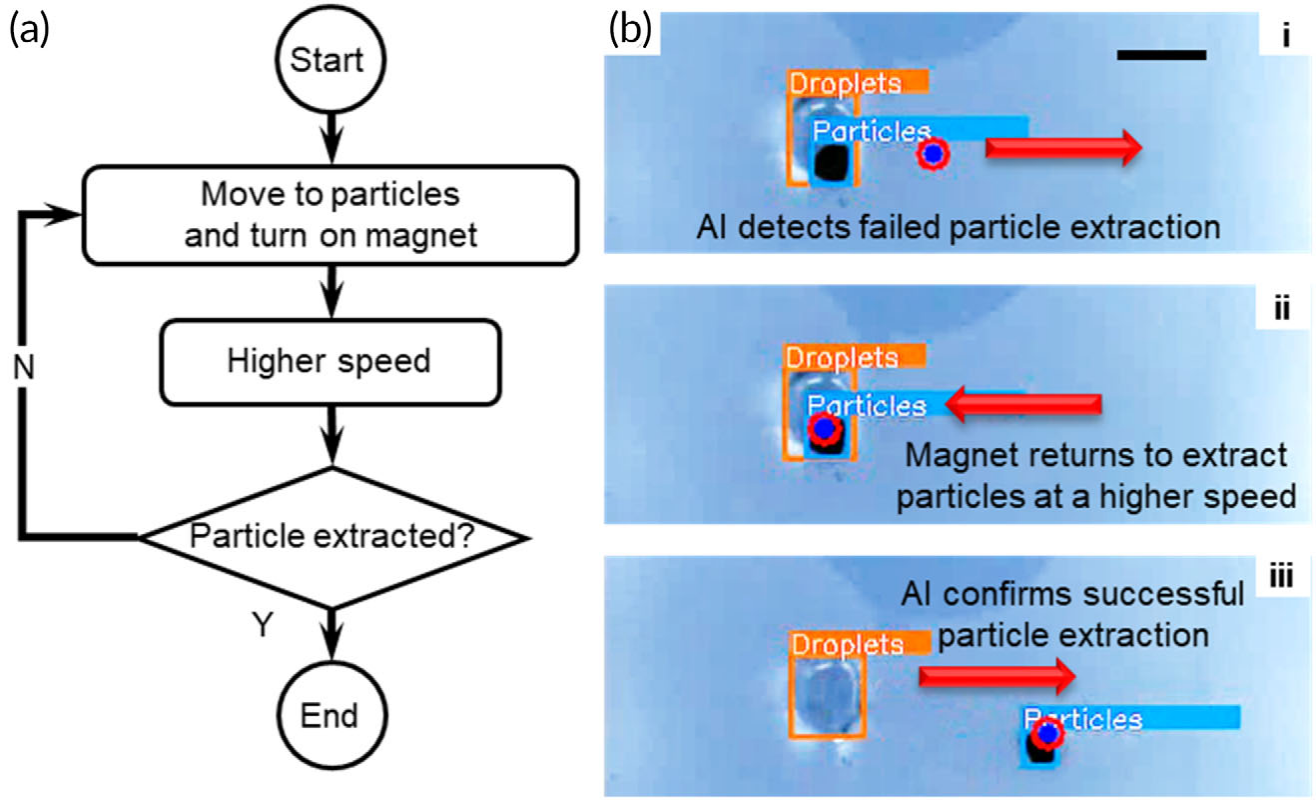
Particle extraction with AI‐empowered image‐based feedback for real‐time closed‐loop control. (a) The flowchart of the control algorithm for particle extraction. (b) Particle extraction with AI‐enabled feedback control. All scale bars represent 5 mm.

Particle extraction is another fundamental operation that separates the solid phase from the liquid phase when performing heterogeneous IVD assays on MDM platforms. The control algorithm for the particle extraction operation is shown in the flowchart in Figure 4a. Before the operation starts, the AI identifies the droplet and particles in live streams, and the CP guides the electromagnet to where the particles are located and switch on the electromagnet. After the user specifies towards which direction the particles are extracted in the GUI, say “EAST,” the CP guides the electromagnet towards right at a high speed to extract particles from the droplet (Figure 4b and Video S2). The particle extraction operation may fail if the quantity of the particles is too large and the moving speed is not high enough.^15^, ^20^ During this operation, the AI monitors the location of the droplet and particles (Figure 4bi). If the two still overlap after the movement, which suggests that the particle extraction operation is unsuccessful, the AI sends a command to bring the electromagnet back to where the particles are and restart the particle extraction process at a higher moving speed (Figure 4bii). Surface energy traps (SETs) may be included to facilitate particle extraction by anchoring the droplet on the substrate.^15^, ^39^ In the end, the AI confirms that the particle extraction operation is successful if the particles and droplet are at two distinctive locations (Figure 4biii). After a successful extraction, a small number of residual particles may be left in the original droplet. However, the amount of these left‐over particles is usually too small to be identified as “Particles” by the AI, and hence they do not affect subsequent operations.

In this operation, AI‐based closed‐loop control again demonstrates the ability to monitor the operation, examine whether it is successfully executed, and rectify the problem if the operation fails, which is unattainable on conventional MDM platforms.

#### 2.2.3 Droplet merging

**FIGURE 5:**
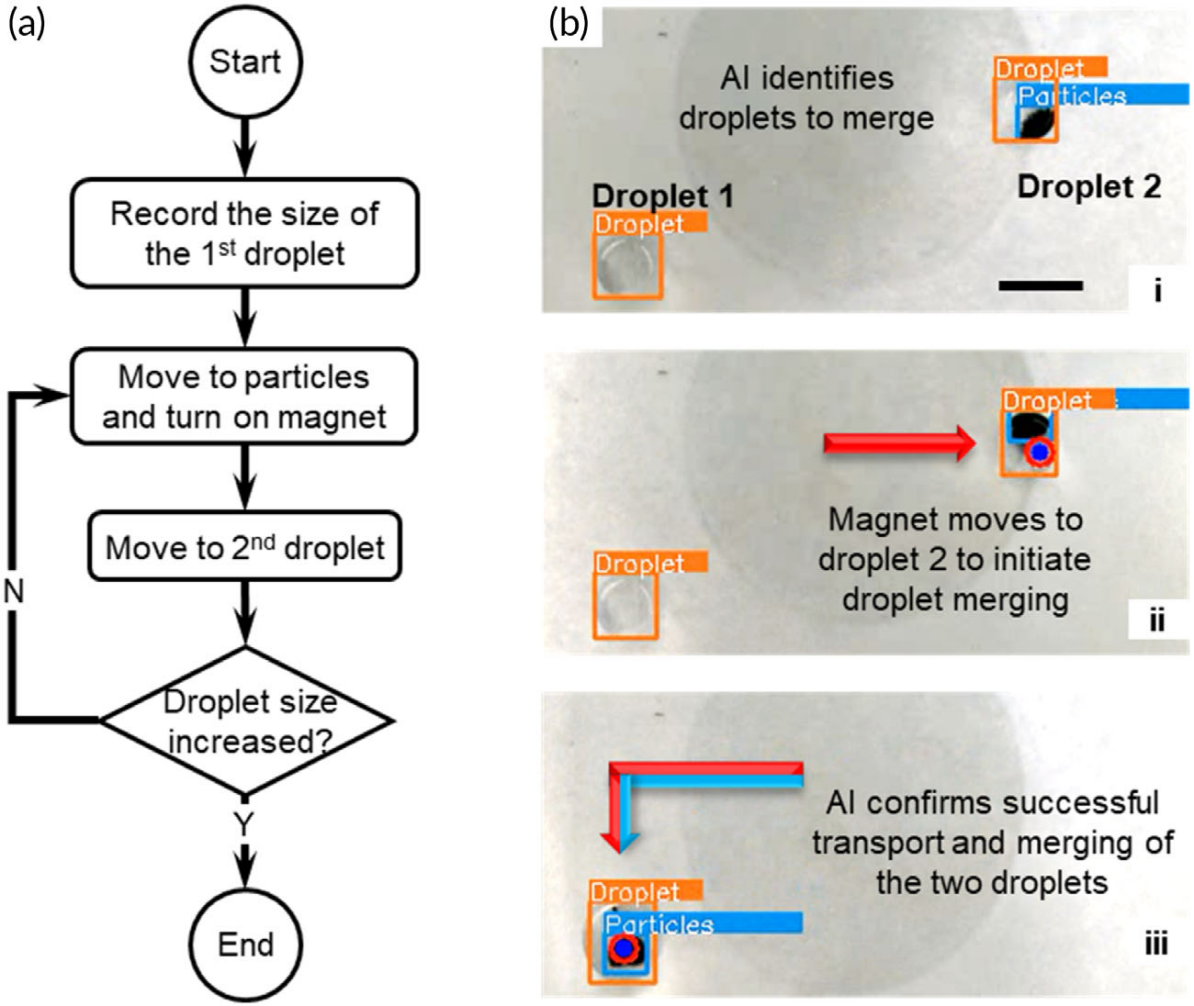
Droplet merging with AI‐empowered image‐based feedback for real‐time closed‐loop control. (a) The flowchart of the control algorithm for droplet merging. (b) Droplet merging with AI‐enabled feedback control. All scale bars represent 5 mm.

Droplet merging is an operation that combines different reactants to initiate bioreactions required for IVD assays on MDM platforms. The control logic for the droplet merging operation is shown in the flowchart in Figure 5a. After the AI identifies the droplet and particles in the live stream, the user is asked to specify which two droplets to merge (Figure 5b and Video S3); the droplet that contains the particles is designated as Droplet 2, and the other one is designated as Droplet 1 (Figure 5bi). The CP guides the electromagnet to Droplet 2 and switches it on (Figure 5bii) to prepare for droplet merging. Next, Droplet 2 is transported to the location of Droplet 1 by the particles and electromagnet. This step is essentially a droplet transport operation, and the “overshoot” function is implemented to bring the center of the droplet close to the destination. Once Droplet 2 reaches the destination, the AI searches the image for the droplet containing particles and compares its size with the original Droplet 1 by measuring the length and width of their bounding boxes. If both the length and width of the bounding box increases by a certain threshold value, typically at least three pixels, the droplet merging operation is deemed a success, and the AI continues with the next operation; otherwise, the CP guides the electromagnet to where the particles are and restart the merging operation.

#### 2.2.4 Passive mixing

**FIGURE 6:**
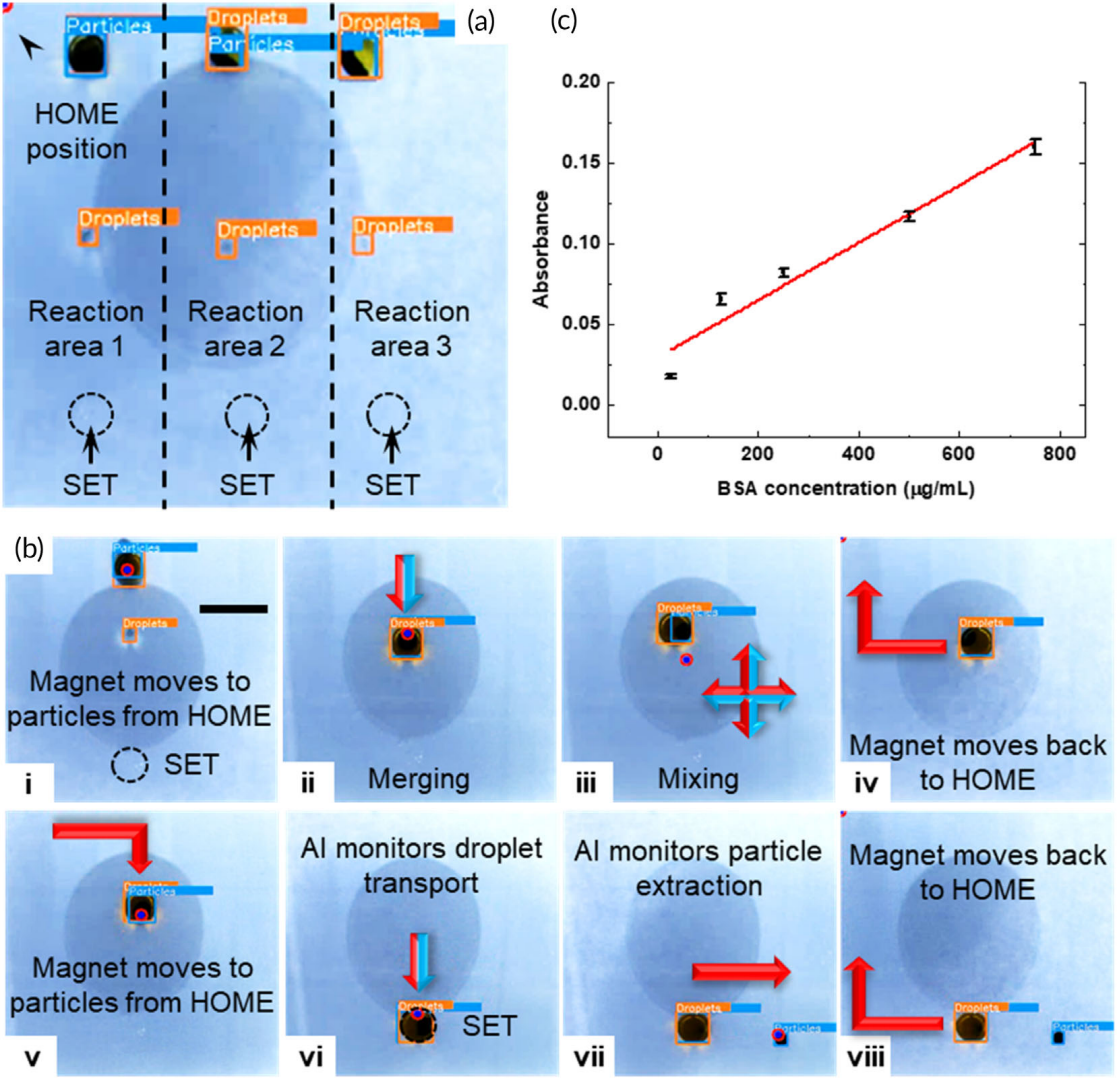
Protein quantification on the AI‐empowered MDM platform. (a) The droplet arrangement for the BCA assay. (b) Droplet operations performed for the BCA assay. (c) The standard curve for BSA quantification on the AI‐empowered MDM platform. All scale bars represent 5 mm.

Once two droplets are merged, the content of the merged droplet is homogenized by moving the particles and droplet on the surface, which promotes passive mixing on the MDM platform.^15^, ^40^ The mixing operation is essentially a combination of a series of droplet transport operations (Figure 6b and Video S4). The user may define customized moving paths for the passive mixing or use the predefined cross‐shaped path with a single click of button. Another parameter that requires user input is the number of loops for mixing. The center of the cross‐shaped path is set to the current location of the particles. The CP guides the electromagnet to move the particles and droplet along each arm of the path and then return to the center. The AI monitors the droplet transport during the mixing operation and determines if the number of remaining loops reaches 0. If yes, the AI ends the operation; otherwise, it starts another loop of mixing.

The fundamental droplet operations shown above demonstrate the unprecedented advantages of the AI‐empowered closed‐loop feedback control for MDM. The ultrafast and light ANN model can analyze images at a speed of 60+ fps on the CPU platform we used, which is fast enough to provide real‐time feedback based on live video recording. The ANN‐based object detector provides the control system with the ability to monitor the droplet location and judge the operation status; these pieces of information are used as the feedback based on which the AI makes decisions on the next move. With this AI‐empowered feedback, checkpoints are put in place to ensure every single operation is performed successfully for automated droplet manipulation on MDM platforms.

### 2.3 IVD on the MDM platform with AI‐empowered real‐time feedback control

#### 2.3.1 Protein quantification

The measurement of serum protein level in biological samples is an important tool for IVD.^41^, ^42^ In this demonstration, bovine serum albumin (BSA) was used as a model protein. The protein quantification by the BCA assay was performed on the AI‐empowered automated MDM platform in triplicates (Figure 6a). The substrate was divided into three reaction areas with one for each replicate. The platform was primed with three 20‐μl droplets of BCA working reagent that contained 1.5 μl of magnetic particles and three 2‐μl droplets of protein solution to be measured. Three SETs were patterned in each reaction area of the substrate to facilitate particle extraction in the last step. Because the relatively strong adhesion of protein solution to the substrate, a relatively large quantity of particles and strong magnetic field (36 V/1.2 A) were used to generate a stronger magnetic force for droplet manipulation. To perform protein quantification, the electromagnet first moved to the location of the particles in Area 1 from the HOME position (Figure 6bi). Next, the working reagent droplet was transported to merge with the protein droplet (Figure 6bii). After merging, the combined droplet was mixed by moving along the cross‐shaped path for five loops and incubated for 10 min (Figure 6biii). During incubation, the electromagnet moved back to the HOME position and was switched off (Figure 6biv). After incubation, the electromagnet moved to the location of the particles and was switched on (Figure 6bv). In the last step, the particles were extracted from the reaction droplet so that the reaction solution could be retrieved from analysis. To perform particle extraction, the reaction droplet was transported to the SET (Figure 6bvi). As the electromagnet drove the particles towards right, the droplet was anchored to the SET, which facilitated particle extraction (Figure 6bvi). After AI confirmed that the droplet and particles were at two distinctive locations, which indicated successful particle extraction, the electromagnet was switched off and returned to the HOME position. To conduct the reaction in triplicates, the CP guided the electromagnet to Areas 2 and 3 in sequence to complete the droplet merging and mixing while the first reaction droplet was being incubated. After incubation, the particle extraction operation was performed sequentially from Areas 1 to 3 to complete the triplicates (Video S4). The protein concentration was determined by measuring the absorbance of the reaction droplet at 480 nm. The same batch of sample was also analyzed in a microwell plate as a benchmark. The standard curve of the absorbance versus the protein concentration is shown in Figure 6c. The results obtained on the AI‐empowered MDM platform agree well with the benchmark (Figure S3). The limit of detection of the MDM‐based assay is 163.2 μg/ml, which is comparable to that of the benchmark of 181.0 μg/ml. Only a single reaction is shown in the figure for clarity. The operation for triplicates is shown in Video S4.

#### 2.3.2 Detection of carbapenemase‐producing Enterobacteriaceae (CPE)

**FIGURE 7:**
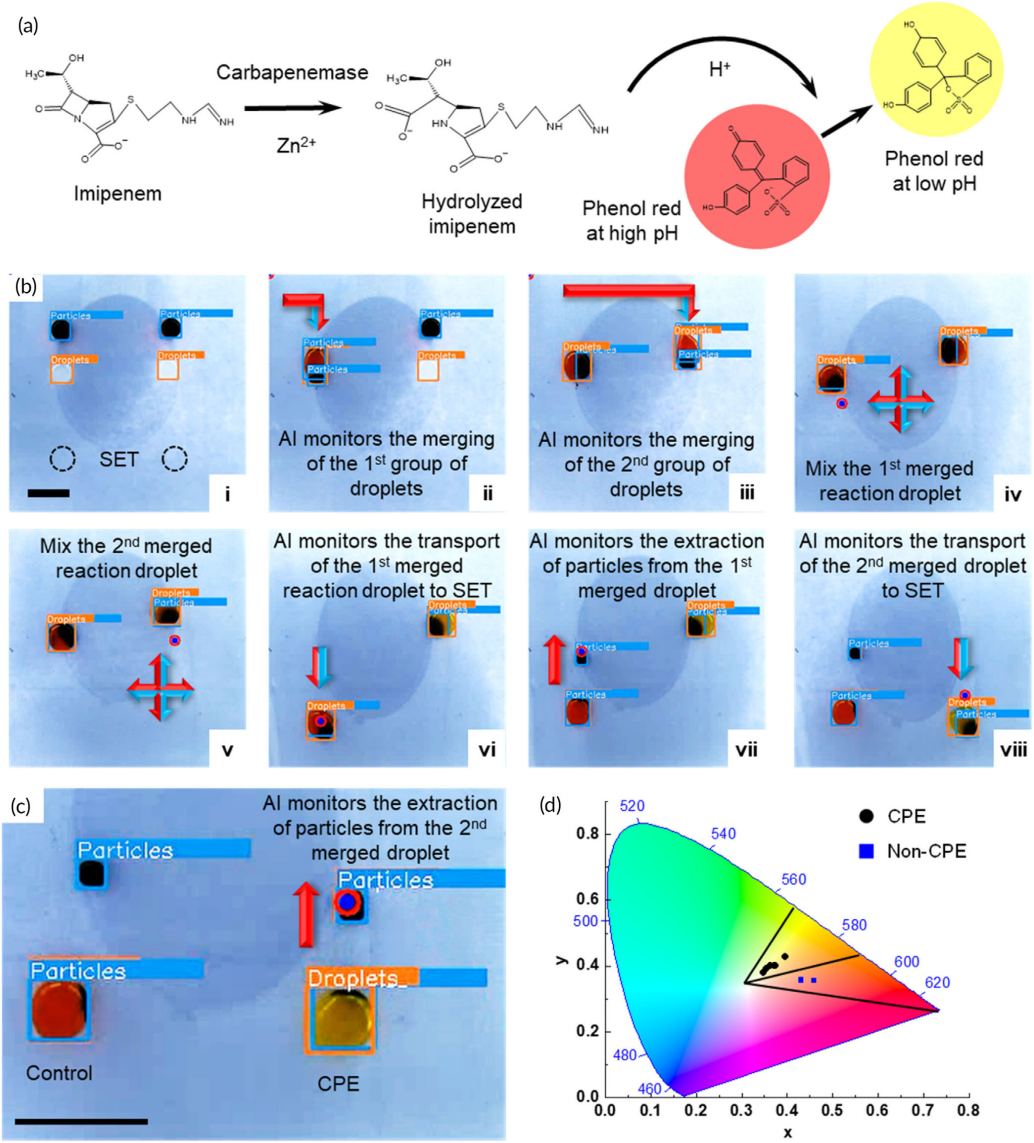
CPE detection on the AI‐empowered MDM platform. (a) Principle of the Carba NP assay. (b) Droplet operations performed for the Carba NP assay. (c) The color‐space mapping of the Carba NP results conducted on the AI‐empowered MDM platform. (d) CPE strains appear in the yellow/orange region, whereas the non‐CPE strain appears in the red region. All scale bars represent 5 mm.

Carbapenem is a group of antibiotics that is considered the last line of defense against Gram negative bacterial infections.^43^ One of the main mechanisms of bacterial resistance to carbapenem is through the production of carbapenemase, an enzyme that hydrolyzes carbapenem. The Carba NP test is a rapid IVD assay that identifies CPE by sensing pH changed induced by the hydrolysis of carbapenem by carbapenemase.^44^, ^45^ In the Carba NP test, each sample is analyzed with a testing reaction that contains imipenem (a type of carbapenem) and a control reaction without imipenem. If the bacterial strain is CPE, it hydrolyzes imipenem in the testing reaction, which reduces the pH and changes the color of the pH indicator from red to yellow/orange (Figure 7a). In contrast, non‐CPE strains are unable to hydrolyze imipenem, and the color of the testing reaction would remain red. In both cases, the color of the control reaction must remain red for the assay to be valid. The Carba NP test on the MDM platform was demonstrated with Strain No. 2 (Table 1) in Figure 7b (Video S5). To perform the Carba NP test on the MDM platform, the substrate was primed with two 10‐μl solution A droplets with 1.5 μl of magnetic particles in each and two 10‐μl droplets containing bacterial lysate. Two SETs were patterned to facilitate particle extraction in the last step (Figure 7bi). First, the electromagnet transported the first solution A droplet (without imipenem) to merge with the bacterial lysate droplet on the left (Figure 7bii), and the same operation was performed to merge the second group of droplets (Figure 7biii). The AI monitored the operation in real time to ensure that the merging operation was completed before moving on to the next step. After merging, the electromagnet moved to the location of the merged droplets and mixed the merged droplets one at a time (Figure 7biv,v). Both the test reaction and control reaction droplets were incubated for 1 h after mixing, during which the imipenem was hydrolyzed if the strain was CPE. To facilitate the observation, the particles were removed from the reaction droplets in the end. To do so, the electromagnet transported the first reaction droplet to a SET where it was anchored to the substrate for easy particle extraction (Figure 7bvi,vii). The same was done for the second reaction droplet (Figure 7bviii,c). Once the particles were extracted, the color of the reaction droplets was observed. Strain No. 2 is a CPE strain that belongs to the KPC type; therefore, the color of the test reaction droplet (on the right) turned into yellow (Figure 7c). The color of the control reaction droplet remained red, indicating that the assay was valid. The RGB values of the droplets in the images were mapped into the color space for objective determination of the droplet color (Figure 7d). A total of eight strains, including six CPE and two non‐CPE strains, were tested on both the AI‐empowered MDM platform and in microwell plate that served as a benchmark (Figure S4). The results obtained with MDM agree with the benchmark, and all eight strains are correctly identified (Table 1).

**TABLE 1:** Summary of Carba NP testing results obtained on the AI‐empowered MDM platform

Sample ID	β‐lactamase type	Species	MDM	Benchmark
2	IMP	*Escherichia coli* NCTC 13476	Positive	Positive
7	KPC	*E. coli* 6013499989	Positive	Positive
8	KPC	*Klebsiella pneumoniae* 6033440078	Positive	Positive
9	IMP	*Serratia marcescens* 6013550755	Positive	Positive
11	NDM	*K. pneumoniae* 2073318014	Positive	Positive
14	NDM	*E. coli* MBRL 235	Positive	Positive
26	None	*K. pneumoniae* C13 (6123‐145679)	Negative	Negative
27	None	*E. coli* C18 (7013‐614848)	Negative	Negative

## 3 MATERIALS AND METHODS

### 3.1 MDM setup

The MDM substrate comprised a piece of glass coverslip of 0.15 mm in thickness. The coverslip was coated with 1% w/w Teflon AF solution (Chemours, USA) diluted in FC 40 (3M, USA). The glass coverslip was mounted onto the customized holders and stages made by 3D printing. A CCD camera (Guardian Vision, China) was mounted above the substrate on an optical rail. A 40 mm × 40 mm region of interest was selected as the field of view. A circular electromagnet (Elecall, China) was mounted on a 2‐axis translational stage with a maximal stroke length of 50 mm (Haijie Jiachuang Corp, China) and placed beneath the substrate. The electromagnet was controlled by an electrical switch (Standard Control Electric, China). A small iron bar with a hemisphere cap of 3‐mm diameter was attached to the center of the electromagnet. When switched on, the electromagnet controlled the motion of the particles and droplets on the substrate via the iron bar. The entire setup was bolted to an optical table (Thorlabs, USA) and placed in a soft‐tent light studio. Three strips of LED light source were fixed to the side wall of the tent for illumination.

### 3.2 ANN model and feedback control system

The ANN object detector used for droplet and particles identification was trained based on Nanodet, an ultrafast and lightweight model optimized for implementation on mobile platforms.^35^ The data set containing 769 images were manually labeled with a total of 3647 labels. All images were converted to the COCO format. The data set was divided into 70%/30% for training/testing so that the training data set contained 538 images and 2604 labels, and the testing data set contained 231 images and 1043 labels. All training parameters were set to default values. COCO mAP (average precision over all classes at IoU = 0.50:0.05:0.95) was calculated to evaluate the performance of the trained models. A GUI was built for users to input control parameters for various droplet operations. The AI made control decisions based on the feedback information and sent commands to the hardware via an Anduino UNO microprocessor.

### 3.3 Fundamental droplet manipulation on the AI‐empowered MDM platform

Silica passivated magnetic particles were used as the droplet actuator (Qiagen, Germany). The default moving speed of the electromagnet was 8.33 mm/s, and the electromagnet was powered by a default power of 24 V/0.8 A. The distance between the electromagnet and the magnetic particles was about 0.2 mm. SETs were created by dispensing a 10‐μl droplet of 95% ethanol and incubating it for 1 h. More ethanol was added during incubation to compensate for the loss due to evaporation. The volumes of the droplet and particles used for the demonstration of droplet transport and merging were 10 and 0.5 μl, respectively. The operation conditions, such as P/D ratio and moving speed, were selected to favor droplet movement. As a result, few failures were encountered when performing droplet movement, and most operations were completed with a single trial. The amount of particles was increased to 1 μl for particle extraction to increase the difficulty of particle extraction so that we could demonstrate how the AI rectify a failed operation. A higher moving speed or the assistance of SETs was used to facilitate the particle extraction in case of failure. In droplet transport, the droplet was considered having reached the destination when the droplet was within a distance of 10 pixels from the destination; in particle extraction, the operation was considered successful when the bounding box of the particles fell outside the bounding box of the droplet; in droplet merging, two droplets were considered merged when the size of the droplet sized increased by at least 3 pixels.

### 3.4 Protein quantification

A BSA serial dilution was quantified using Pierce™ Rapid Gold BCA Protein Assay Kit (Thermo science, A53225, USA) by following manufacturer's instruction. The volumes of the working reagent were 200 μl for the benchmark measurement in microwell plates and 20 μl in droplets on the MDM platform. All other samples and reagents were scaled proportionally. 1.5 μl of magnetic particles were added for droplet actuation. The absorbance at 480 m was measured by a UV–Vis spectrometer (Biochrom, USA).

### 3.5 Carba NP test

Detailed protocol for bacteria subculture and the preparation of Carba NP reagents were reported in our previous work.^16^, ^46^ All chemical reagents were purchased from Sigma‐Aldrich unless otherwise stated. Bacterial strains were provided by the National Center for Infectious Diseases (NCID) of Singapore and Mayo Clinic (Rochester, Minnesota, USA), and their resistance was determined by molecular tests. Six strains were CPE, including 1 VIM, 1 KPC, 1 KEP, 1 IMP and 2 NDM subtypes; two strains were non‐CPE. To perform Carba NP, a 1‐μl loopful of bacterial colony was picked from the culture plate and inoculated in 100 μl of bacterial lysis buffer (bacterial protein extraction reagent in Tris). The entire volume of bacterial lysate was used for the benchmark measurement, whereas 10 μl was used for the MDM measurement. Solution A consisted of 0.5% w/v of phenol red in 10 mM zinc sulfate buffered to pH 7.8 with 0.1 N NaOH. The testing reagent contained solution A with 12 mg/ml of imipenem/cilastatin (Fresenius Kabi, Germany) while the control reagent only contained solution A.

## 4 CONCLUSION

In conclusion, we have developed an AI‐empowered automated MDM platform that uses an ultrafast and lightweight ANN object detector to identify droplets and particles for real‐time closed‐loop feedback control. The AI‐empowered MDM platform shows the ability to rectify failed droplet operations by monitoring the location of droplet and magnetic particles and judging the operation status. Its ability to conduct fully automated IVD is demonstrated with droplet‐based protein quantification and Carba NP assay for antibiotic resistance detection. In both cases, the results obtained on the MDM platform agree with the benchmarks. We envision that this AI‐based feedback approach will be widely adopted not only by MDM but also by other types of digital microfluidic platforms to offer precise and error‐free droplet operations for a wide range of automated IVD applications. Furthermore, colorimetric readout could also be integrated with the image‐based object detector in the future by training the ANN models to determine the color and intensity of the reaction droplets. By doing so, we can build a more powerful smart system with both actuation and sensing capability powered by AI.

Nonetheless, this study is a preliminary proof‐of‐concept, and we notice several limitations with the current implementation. The main issue we have faced is caused by the misidentification of droplets. This is particularly problematic for small transparent droplets that frequently suffer from misdetection, in which case the AI is unable to make the correct decision. A potential solution to this problem is to improve the illumination conditions so that the droplet features are more prominent in the images. Another limitation is the relatively small data set used for training. Increasing the size of the training data set by including more droplet‐particle combinations will improve the mAP of the ANN‐based object detector for more accurate feedback control.

## AUTHOR CONTRIBUTIONS

**Yuxuan Tang:** Data curation (lead); formal analysis (lead); investigation (equal); methodology (lead); software (lead); visualization (lead); writing – original draft (equal); writing – review and editing (equal). **Fei Duan:** Conceptualization (supporting); investigation (equal); methodology (supporting); project administration (equal); supervision (equal); writing – original draft (equal); writing – review and editing (equal). **Aiwu Zhou:** Data curation (equal); formal analysis (supporting); methodology (supporting); software (supporting); validation (equal); writing – review and editing (supporting). **Pojchanun Kanittamniyom:** Data curation (equal); formal analysis (equal); methodology (supporting); resources (equal); validation (equal); writing – review and editing (supporting). **Shaobo Luo:** Formal analysis (supporting); methodology (equal); software (equal); writing – review and editing (supporting). **Xuyang Hu:** Data curation (equal); methodology (supporting); resources (supporting); writing – review and editing (supporting). **Xudong Jiang:** Formal analysis (supporting); methodology (equal); software (supporting); writing – review and editing (supporting). **Shawn Vasoo:** Methodology (supporting); resources (equal); writing – review and editing (supporting). **Xiaosheng Zhang**: Conceptualization (equal); formal analysis (equal); funding acquisition (lead); project administration (equal); resource (equal); supervision (equal); writing ‐ review and editing (equal). **Yi Zhang:** Conceptualization (lead); formal analysis (equal); funding acquisition (equal); investigation (equal); methodology (equal); project administration (lead); resources (lead); supervision (lead); visualization (lead); writing – original draft (equal); writing – review and editing (lead).

## CONFLICT OF INTEREST

Yi Zhang declares equity interest in DropLab Scientific (Singapore) Pvt. Ltd and Guangzhou DropLab Scientific Co., Ltd. The rest authors declare no conflict of interest.

## Supporting information


**Data S1:** Supporting Information.

Video S1:

Video S2:

Video S3:

Video S4:

Video S5:

## Data Availability

Data available on request from the authors.

## References

[1] Rohr U‐P , Binder C , Dieterle T , et al. The value of in vitro diagnostic testing in medical practice: a status report. PLoS One. 2016;11(3):e0149856. 26942417 10.1371/journal.pone.0149856 PMC4778800

[2] La Marca A, Capuzzo M, Paglia T, Roli L, Trenti T, Nelson SM. Testing for SARS‐CoV‐2 (COVID‐19): a systematic review and clinical guide to molecular and serological in‐vitro diagnostic assays. Reprod Biomed Online. 2020;41(3):483‐499. 32651106 10.1016/j.rbmo.2020.06.001 PMC7293848

[3] Vashist SK . In vitro diagnostic assays for COVID‐19: recent advances and emerging trends. Diagnostics. 2020;10(4):202. 32260471 10.3390/diagnostics10040202 PMC7235801

[4] Lim SM, Cheng HL, Jia H, et al. Finger stick blood test to assess post vaccination SARS‐CoV‐2 neutralizing antibody response against variants. Bioeng Transl Med. 2022;7(2):e10293. 35600666 10.1002/btm2.10293 PMC9115707

[5] Wang YC , Lee YT , Yang T , Sun JR , Shen CF , Cheng CM . Current diagnostic tools for coronaviruses—From laboratory diagnosis to POC diagnosis for COVID‐19. Bioeng Transl Med. 2020;5(3):e10177. 32838038 10.1002/btm2.10177 PMC7435577

[6] Zhang Y , Rogers A , Nadeau K , Gu J , Yang S . A perspective on the role of point‐of‐care “immuno‐triaging” to optimize COVID‐19 vaccination distribution in a time of scarcity. Front Public Health. 2021;9:638316. 34414149 10.3389/fpubh.2021.638316 PMC8369238

[7] Samiei E , Tabrizian M , Hoorfar M . A review of digital microfluidics as portable platforms for lab‐on a‐chip applications. Lab Chip. 2016;16(13):2376‐2396. 27272540 10.1039/c6lc00387g

[8] Geng H , Feng J , Stabryla LM , Cho SK . Dielectrowetting manipulation for digital microfluidics: creating, transporting, splitting, and merging of droplets. Lab Chip. 2017;17(6):1060‐1068. 28217772 10.1039/c7lc00006e

[9] Zhang Y , Nguyen N‐T . Magnetic digital microfluidics–a review. Lab Chip. 2017;17(6):994‐1008. 28220916 10.1039/c7lc00025a

[10] Zhang Y . Magnetic digital microfluidics for point‐of‐care testing: where are we now? Curr Med Chem. 2021;28(31):6323‐6336. 32881657 10.2174/0929867327666200903115448

[11] Anderson S , Hadwen B , Brown C . Thin‐film‐transistor digital microfluidics for high value in vitro diagnostics at the point of need. Lab Chip. 2021;21(5):962‐975. 33511381 10.1039/d0lc01143f

[12] Pollack MG , Pamula VK , Srinivasan V , Eckhardt AE . Applications of electrowetting‐based digital microfluidics in clinical diagnostics. Expert Rev Mol Diagn. 2011;11(4):393‐407. 21545257 10.1586/erm.11.22

[13] Choi K , Ng AHC , Fobel R , Wheeler AR . Digital microfluidics. Annu Rev Anal Chem. 2012;5:413‐440. 10.1146/annurev-anchem-062011-143028 22524226

[14] Ding X , Li P , Lin SCS , et al. Surface acoustic wave microfluidics. Lab Chip. 2013;13(18):3626‐3649. 23900527 10.1039/c3lc50361e PMC3992948

[15] Zhang Y , Wang TH . Full‐range magnetic manipulation of droplets via surface energy traps enables complex bioassays. Adv Mater. 2013;25(21):2903‐2908. 23529938 10.1002/adma.201300383 PMC3964134

[16] Kanitthamniyom P , Zhou A , Feng S , Liu A , Vasoo S , Zhang Y . A 3D‐printed modular magnetic digital microfluidic architecture for on‐demand bioanalysis. Microsyst Nanoeng. 2020;6(1):1‐11. 34567660 10.1038/s41378-020-0152-4 PMC8433373

[17] Pipper J , Zhang Y , Neuzil P , Hsieh TM . Clockwork PCR including sample preparation. Angew Chem Int Ed. 2008;47(21):3900‐3904. 10.1002/anie.200705016 18412211

[18] Kanitthamniyom P , Zhang Y . Magnetic digital microfluidics on a bioinspired surface for point‐of‐care diagnostics of infectious disease. Electrophoresis. 2019;40(8):1178‐1185. 10.1002/elps.201900074 30770588

[19] Pipper J , Inoue M , Ng LFP , Neuzil P , Zhang Y , Novak L . Catching bird flu in a droplet. Nat Med. 2007;13(10):1259‐1263. 17891145 10.1038/nm1634 PMC7095864

[20] Long Z , Shetty AM , Solomon MJ , Larson RG . Fundamentals of magnet‐actuated droplet manipulation on an open hydrophobic surface. Lab Chip. 2009;9(11):1567‐1575. 19458864 10.1039/b819818g PMC2932710

[21] Zhang Y , Zhou A , Chen S , Lum GZ , Zhang X . A perspective on magnetic microfluidics: towards an intelligent future. Biomicrofluidics. 2022;16(1):011301. 35069962 10.1063/5.0079464 PMC8769766

[22] Shih SC, Fobel R, Kumar P, Wheeler AR . A feedback control system for high‐fidelity digital microfluidics. Lab Chip. 2011;11(3):535‐540. 21038034 10.1039/c0lc00223b

[23] Gong J . All‐electronic droplet generation on‐chip with real‐time feedback control for EWOD digital microfluidics. Lab Chip. 2008;8(6):898‐906. 18497909 10.1039/b717417a PMC2769494

[24] Ren H , Fair RB , Pollack MG . Automated on‐chip droplet dispensing with volume control by electro‐wetting actuation and capacitance metering. Sens Actuators B. 2004;98(2–3):319‐327.

[25] Khorshidi MA , Rajeswari PKP , Wählby C , Joensson HN , Andersson Svahn H . Automated analysis of dynamic behavior of single cells in picoliter droplets. Lab Chip. 2014;14(5):931‐937. 24385254 10.1039/c3lc51136g

[26] Jin K, Huang Q, Hu C, Hu S, Li J. A digital microfluidic system with integrated electrochemical impedance detection arrays. J Phys: Conf Ser. 2022;2196:012005.

[27] Jain V , Patrikar RM . A low‐cost portable dynamic droplet sensing system for digital microfluidics applications. IEEE Trans Instrum Meas. 2020;69(6):3623‐3630.

[28] Zantow M , Dendere R , Douglas TS . Image‐Based Analysis of Droplets in Microfluidics. in 2013 35th Annual International Conference of the IEEE Engineering in Medicine and Biology Society (EMBC) . 2013. IEEE. 10.1109/EMBC.2013.6609865 24110052

[29] Vo PQN, Husser MC, Ahmadi F, Sinha H, Shih SCC . Image‐based feedback and analysis system for digital microfluidics. Lab Chip. 2017;17(20):3437‐3446. 28871290 10.1039/c7lc00826k

[30] Luo Z , Huang B , Xu J , et al. Machine vision‐based driving and feedback scheme for digital microfluidics system. Open Chem. 2021;19(1):665‐677.

[31] Li L , Gu Z , Zhou JL , et al. Intelligent droplet tracking with correlation filters for digital microfluidics. Chin Chem Lett. 2021;32(11):3416‐3420.

[32] Lashkaripour A , Rodriguez C , Mehdipour N , et al. Machine learning enables design automation of microfluidic flow‐focusing droplet generation. Nat Commun. 2021;12(1):1‐14. 33397940 10.1038/s41467-020-20284-z PMC7782806

[33] Hettiarachchi S , Melroy G , Mudugamuwa A , et al. Design and development of a microfluidic droplet generator with vision sensing for lab‐on‐a‐chip devices. Sens Actuat A: Phys. 2021;332:113047.

[34] Momtahen S , Al‐Obaidy F , Mohammadi F . Machine learning with digital microfluidics for drug discovery and development. in 2019 IEEE Canadian Conference of Electrical and Computer Engineering (CCECE) . 2019. IEEE.

[35] RangiLyu, NanoDet . 2020: p. Github v0.1.0.

[36] Tian Z , Shen C , Chen H , He T. FCOS: Fully Convolutional One‐Stage Object Detection. Paper presented at: 2019 IEEE/CVF International Conference on Computer Vision (ICCV). October 27 ‐ November 02, 2019; Seoul, Korea.

[37] Zhang S. , Chi C , Yao Y , et al. Bridging the gap between anchor‐based and anchor‐free detection via adaptive training sample selection. Proceedings of the IEEE/CVF conference on computer vision and pattern recognition, Seattle, WA, 13‐19 June 2020. IEEE; 2020.

[38] Li X , Wang W, Wu L, et al., Generalized focal loss: learning qualified and distributed bounding boxes for dense object detection . arXiv preprint arXiv:2006.04388, 2020.

[39] Zhang Y , Shin DJ , Wang T‐H . Serial dilution via surface energy trap‐assisted magnetic droplet manipulation. Lab Chip. 2013;13(24):4827‐4831. 24162777 10.1039/c3lc50915j PMC3963271

[40] Kanitthamniyom P , Hon PY , Zhou A , et al. A 3D‐printed magnetic digital microfluidic diagnostic platform for rapid colorimetric sensing of carbapenemase‐producing Enterobacteriaceae. Microsyst Nanoeng. 2021;7(1):47. 34567760 10.1038/s41378-021-00276-9 PMC8433351

[41] O'Connell T , Horita TJ , Kasravi B . Understanding and interpreting the serum protein electrophoresis. Am Fam Physician. 2005;71(1):105‐112. 15663032

[42] Tothova C , Nagy O , Kovac G . Serum proteins and their diagnostic utility in veterinary medicine: a review. Vet Med. 2016;61(9):475‐496.

[43] Codjoe FS , Donkor ES . Carbapenem resistance: A review. Med Sci. 2018;6(1):1. 10.3390/medsci6010001 PMC5872158 29267233

[44] Nordmann P , Poirel L , Dortet L . Rapid detection of carbapenemase‐producing Enterobacteriaceae. Emerg Infect Dis. 2012;18(9):1503‐1507. 22932472 10.3201/eid1809.120355 PMC3437707

[45] Dortet L , Poirel L , Nordmann P . Rapid detection of carbapenemase‐producing Pseudomonas spp. J Clin Microbiol. 2012;50(11):3773‐3776. 22972829 10.1128/JCM.01597-12 PMC3486216

[46] Zhou A, Xu C, Kanitthamniyom P , et al. Magnetic soft millirobots 3D printed by circulating vat photopolymerization to manipulate droplets containing hazardous agents for in vitro diagnostics. Adv Mater. 2022;34(15):2200061. 10.1002/adma.202200061 35147257

