# Supplementary material for: Image‐based real‐time feedback control of magnetic digital microfluidics by artificial intelligence‐empowered rapid object detector for automated in vitro diagnostics
Source: Bioeng Transl Med. 2022 Oct 18;8(4):e10428. doi: 10.1002/btm2.10428 (PMC10354763; doi:10.1002/btm2.10428)
Supplement: Supplementary file 1 — Data S1: Supporting Information. [file BTM2-8-e10428-s002.docx]

**Supporting Information for Image-Based Real-Time Feedback Control of Magnetic Digital Microfluidics by Artificial Intelligence-Empowered Rapid Object Detector for Automated *In Vitro* Diagnostics**

Yuxuan Tang^1^, Fei Duan^1^, Aiwu Zhou^2^, Pojchanun Kanitthamniyom^1^, Shaobo Luo^3^, Xuyang Hu^4^, Xudong Jiang^5^, Shawn Vasoo^6^, Xiaosheng Zhang^7^*, and Yi Zhang^7^*

^1^  School of Mechanical and Aerospace Engineering, Nanyang Technological University

^2^ Singapore Center for 3D Printing, School of Mechanical and Aerospace Engineering, Nanyang Technological University

^3^ School of Microelectronics, Southern University of Science and Technology

^4^ China-Singapore International Joint Research Institute

^5^ School of Electronic and Electrical Engineering, Nanyang Technological University

^6^ National Center for Infectious Disease, Tan Tock Seng Hospital

^7^ School of Electronic Science and Engineering, University of Electronic Science and Technology of China

* Correspondence should be addressed to Yi Zhang [yi_zhang@uestc.edu.cn](mailto:yi_zhang@uestc.edu.cn) and Xiaosheng Zhang [xszhang@uestc.edu.cn](mailto:xszhang@uestc.edu.cn)


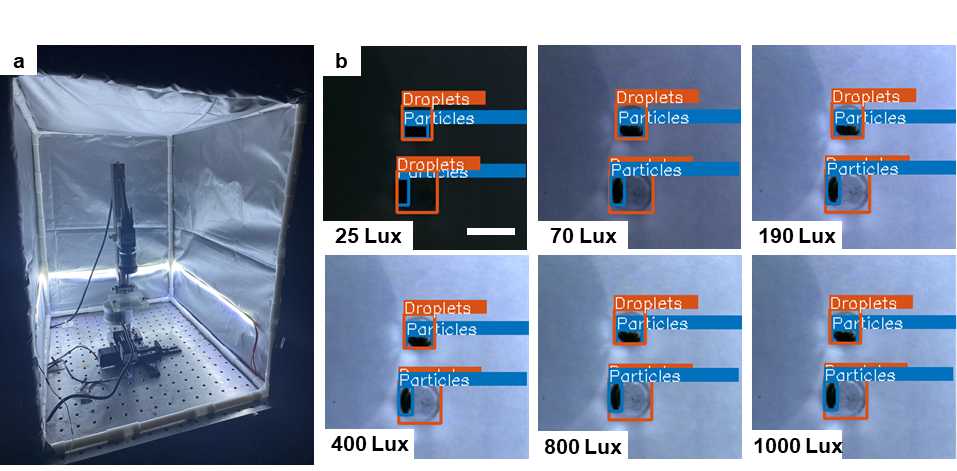


**Fig. S1** d) AI-empowered MDM setup: a soft-tent light studio equipped with three strips of intensity-adjustable LED illumination source; e) Droplets and particles are successfully identified under a wide range of illumination conditions.


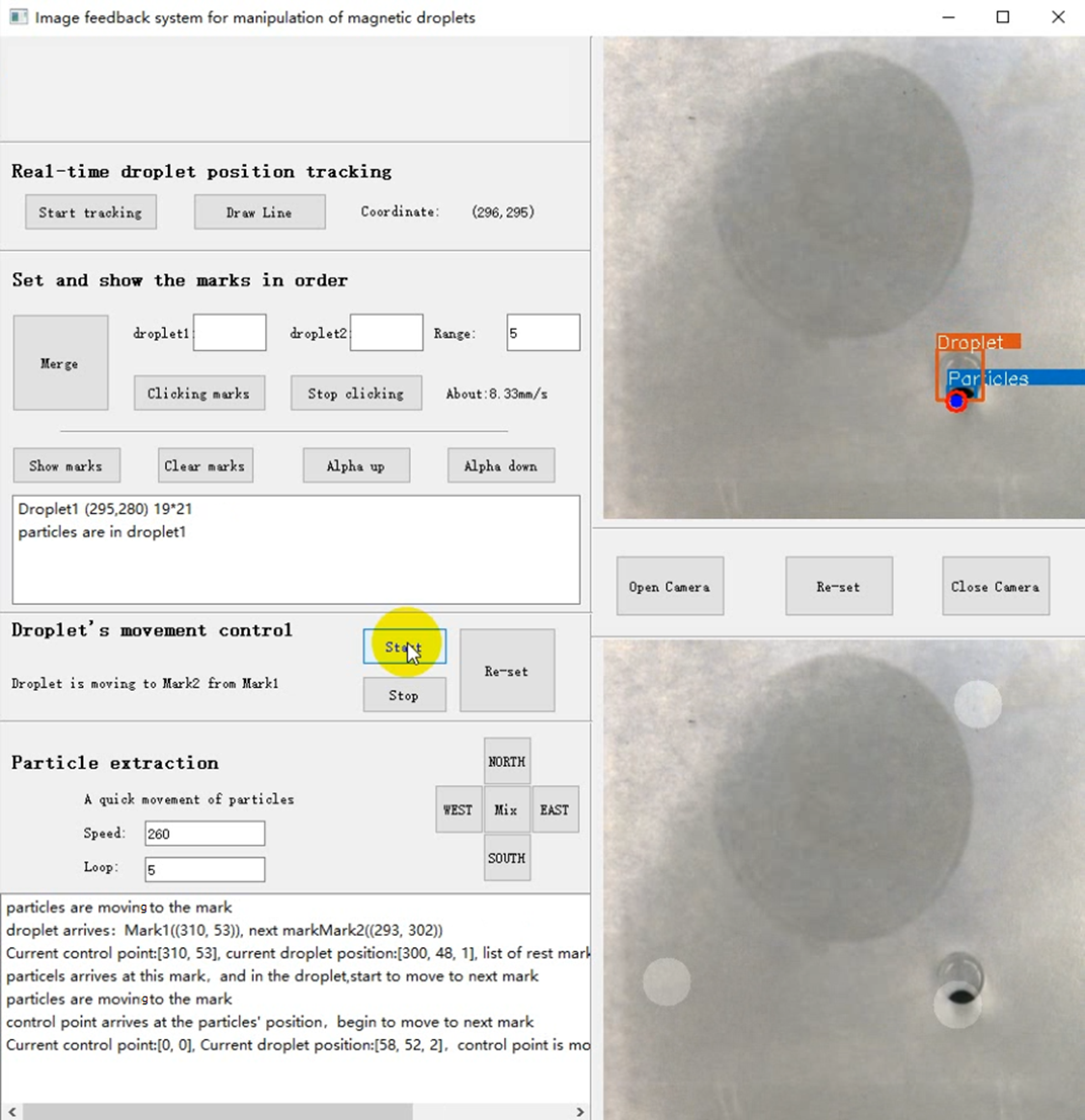


**Fig. S2** The graphic user interface.


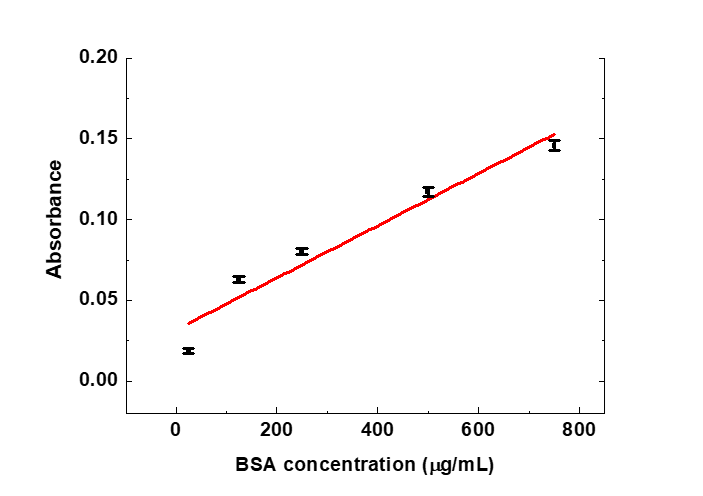


**Fig. S3** The standard curve of BSA quantification based on the results obtained in microwell plate.


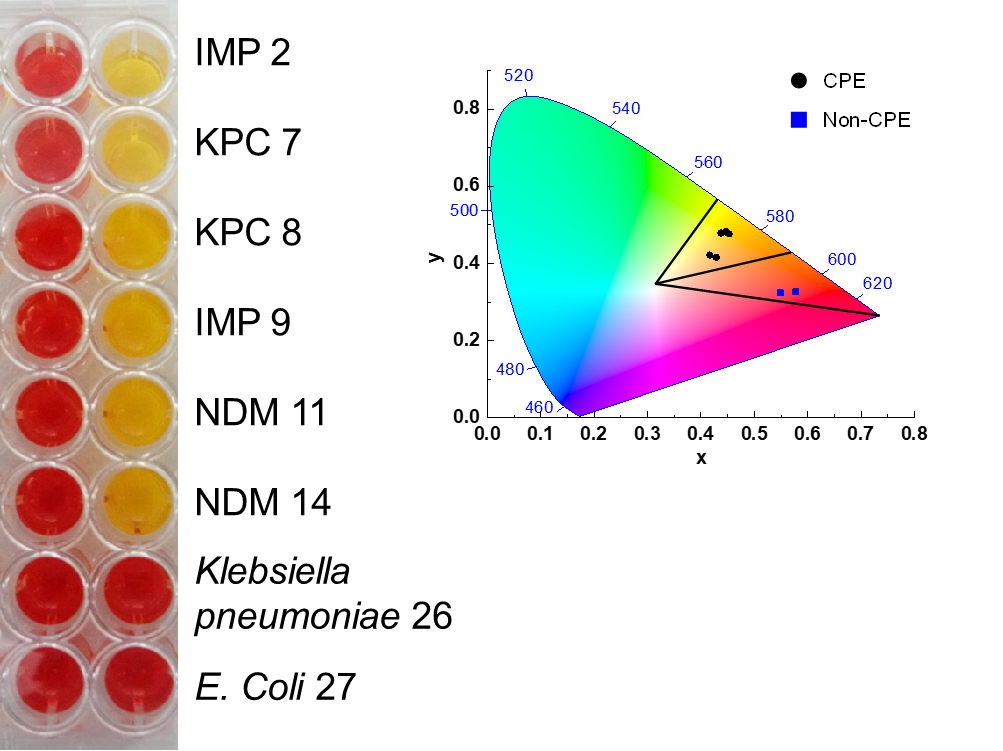


**Fig. S4** Benchmark Carba NP performed in a microwell plate along with the results mapped into the color space.

**Pseudocode of Droplet Operations**

**Droplet transport:**

Add marks by clicking or dragging cursor to set a path for droplet's movement

Start transport function

CP moves to MP's current position

i = 1

While i <= the amount of marks

CP moves to Mark.i

If distance between droplet and Mark.i <= 10 pixels

i ← i + 1

Else

While droplet does not arrive at Mark.i

CP moves to MP's current position

CP moves to Mark.i

End if

Endwhile

End

---------------------------------------------------------------------------------------------------------------------------------

**Particle extraction:**

Start extraction function

CP moves to MP's current position

CP moves 60 pixels in one direction

While MP is in droplet

Raise CP's moving speed

CP moves to MP's current position

CP moves 60 pixels in one direction

Endwhile

End

---------------------------------------------------------------------------------------------------------------------------------

**Droplet merging:**

Input two droplets' serial numbers, 1st droplet should have MP

Start droplet merging

CP moves to MP's current position

CP moves to 2nd droplet

While droplet which contain MP becomes bigger

CP moves to the 2nd droplet

Endwhile

End

---------------------------------------------------------------------------------------------------------------------------------

**Passive mixing:**

Input mixing's times in variable l

Start mixing

While l > 0

CP cross movement

l ← l - 1

Endwhile

End
